# 2219: Amyotrophic lateral sclerosis, stem cells and TALENted technology

**DOI:** 10.1017/cts.2017.212

**Published:** 2018-05-10

**Authors:** Shaheen Kurani, Nicolas Madigan, Karl Clark, Stephen Ekker, Nathan Staff, Anthony Windebank

## Abstract

OBJECTIVES/SPECIFIC AIMS: The current treatment for amyotrophic lateral sclerosis (ALS) includes systemic delivery of neurotrophic factors (NTFs). Although this approach may seem theoretically sound, NTF efficacy within the central nervous system (CNS) is largely limited by the blood-brain barrier. Thus, a cell-based approach, which allows for targeted delivery of molecular therapies locally from the CNS, could lead to a paradigm shift in the field. METHODS/STUDY POPULATION: The Windebank and Staff group at Mayo Clinic completed a Phase I dose-escalation safety trial of autologous, adipose-derived mesenchymal stem cells (adMSCs) in an effort to move toward personalized medical treatment of ALS. The adMSCs were injected into the intrathecal space by lumbar puncture in 27 patients and the results showed an excellent safety profile across a range of doses. The team is moving forward with this idea by using gene-editing technology to develop clinical-grade, genetically modified autologous MSCs. The patient-derived adMSCs are modified at defined “safe-harbor” regions of the human genome through transcription activator-like effector nuclease (TALEN) technology. RESULTS/ANTICIPATED RESULTS: Our results show that electroporating adMSCs with plasmid DNA leads to efficient GFP or TALEN transgene expression, but yields low cell survival and a low rate of genetic modification. DISCUSSION/SIGNIFICANCE OF IMPACT: It can be concluded that: (1) TALEN technology may be used to target safe harbor loci for gene integration to produce therapeutic adMSC for ALS. (2) Primary barriers to adMSC modification are inefficient TALEN and donor template uptake, low cutting efficiency, and poor cell survival after electroporation. Future directions include optimizing the protocol to obtain 48 base pairs in the homology arms and increasing transfection efficiency.

